# Increased *Leishmania infantum* resistance to miltefosine and amphotericin B after treatment of a dog with miltefosine and allopurinol

**DOI:** 10.1186/s13071-021-05100-x

**Published:** 2021-12-09

**Authors:** Gustavo Gonçalves, Monique Paiva Campos, Alessandra Silva Gonçalves, Lia Carolina Soares Medeiros, Fabiano Borges Figueiredo

**Affiliations:** 1 Cell Biology Laboratory, Carlos Chagas Institute, Oswaldo Cruz Foundation (FIOCRUZ), Curitiba, Paraná 81310-020 Brazil; 2 Private Practice, Campo Grande, Brazil

**Keywords:** Amastigote, Clinical isolate, In vitro test, Promastigote

## Abstract

**Background:**

*Leishmania infantum* is the most important etiological agent of visceral leishmaniasis in the Americas and Mediterranean region, and the dog is the main host. Miltefosine was authorized to treat canine leishmaniasis (CanL) in Brazil in 2017, but there is a persistent fear of the emergence of parasites resistant not only to this drug but, through cross-resistance mechanisms, also to meglumine antimoniate and amphotericin B. Additionally, the literature shows that acquisition of resistance is followed by increased parasite fitness, with higher rates of proliferation, infectivity and metacyclogenesis, which are drivers of parasite virulence. In this context, the aim of this study was to analyze the impact of treating a dog with miltefosine and allopurinol on the generation of parasites resistant to miltefosine, amphotericin B and meglumine antimoniate.

**Methods:**

In vitro susceptibility tests were conducted against miltefosine, amphotericin B and meglumine antimoniate with T0 (parasites isolated from a dog before treatment with miltefosine plus allopurinol), T1 (after 1 course of treatment) and T2 (after 2 courses of treatment) isolates. The rates of cell proliferation, infectivity and metacyclogenesis of the isolates were also evaluated.

**Results:**

The results indicate a gradual increase in parasite resistance to miltefosine and amphotericin B with increasing the number of treatment courses. An increasing trend in the metacyclogenesis rate of the parasites was also observed as drug resistance increased.

**Conclusion:**

The data indicates an increased *L. infantum* resistance to miltefosine and amphotericin B after the treatment of a dog with miltefosine plus allopurinol. Further studies with a larger number of *L. infantum* strains isolated from dogs with varied immune response profiles and undergoing different treatment regimes, are advocated.

**Graphical Abstract:**

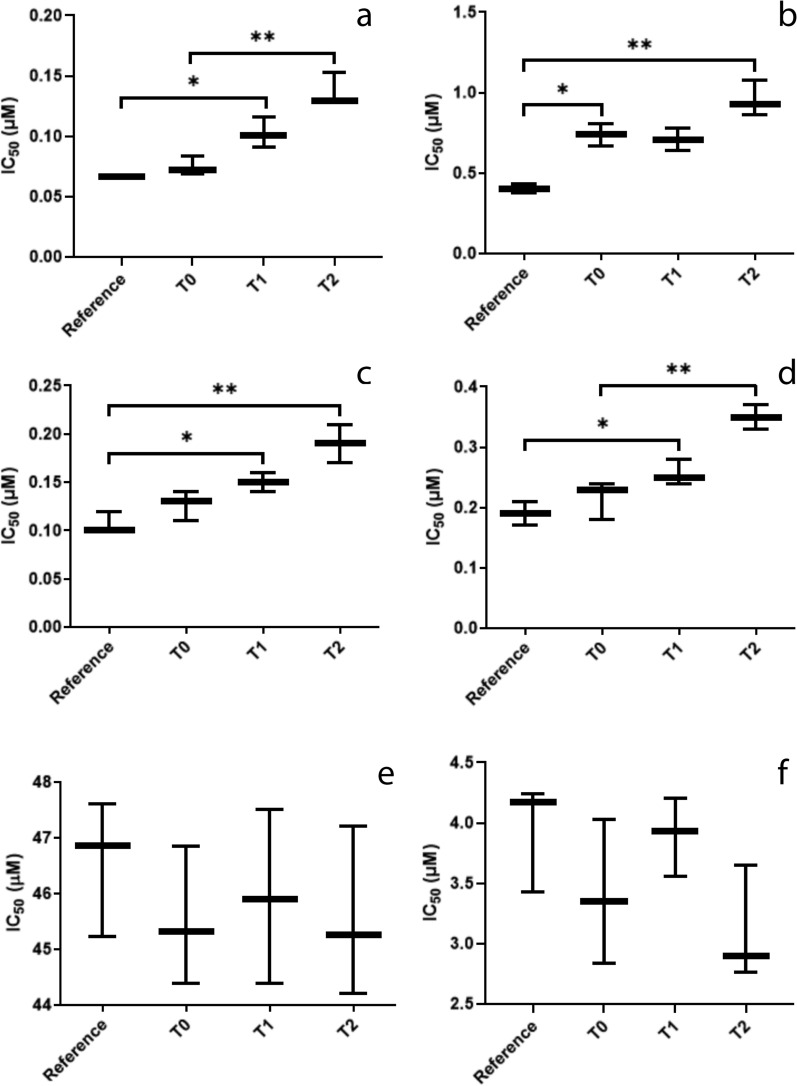

*Leishmania infantum* is the most important etiological agent of visceral leishmaniasis (VL) in the Americas and the Mediterranean region [1], and the dog is the main host [2]. Canine leishmaniasis (CanL) usually precedes the occurrence of human cases [2], and control of the zoonotic cycle remains a challenge [3].

In 2017, Brazilian regulatory agencies authorized the use of miltefosine to treat canine leishmaniasis (CanL) in Brazil [4], and it remains the only treatment for CanL currently available in the country. Despite therapeutic success in most cases [5], treatment failures have been observed in both monotherapy and combined therapies [6], with improvement in canine clinical signs not always followed by parasite clearance [7]. Thus, miltefosine treatment is still not considered a fully effective control measure because in addition to the risk of parasite resistance, relapses are reported and dogs can continue to infect the invertebrate host even weeks after the end of treatment, despite being clinically cured [8]. For this reason, a fundamental part of CanL treatment is to use insecticide-impregnated collars on dogs undergoing treatment.

In addition to the treatment of dogs with miltefosine being associated with the possible emergence of parasites resistant to this drug, some in vitro studies have shown cross-resistance to other drugs [9, 10] that can lead to the emergence of parasites resistant not only to miltefosine, but also to other drugs used to treat VL. Moreover, there are also reports on the impact of acquisition of resistance on parasite fitness, in which drug-resistant parasites presented higher rates of cell proliferation, metacyclogenesis and infectivity compared with those of susceptible parasites [11–13], which are aggravating factors of disease virulence [14].

In this context, the aim of the present study was to analyze the impact of treating a dog with CanL with miltefosine plus allopurinol on the generation of parasites resistant to miltefosine, amphotericin B and meglumine antimoniate. A secondary aim was to determine the impact of the possible acquisition of resistance on the rates of cell proliferation, metacyclogenesis and infectivity of the parasite.

The isolates used in this study were obtained from a naturally infected, mixed-breed female dog, aged approximately 5 years, from the municipality of Campo Grande, state of Mato Grosso do Sul, Brazil. After positive serological diagnosis using the Dual-path Platform chromatographic immunoassay (DPP®), additional collections were performed to confirm the infection by *L. infantum* through quantitative PCR (qPCR) and parasitological culture. For the qPCR, a 3-mm-diameter intact skin fragment of the dog was obtained by punch biopsy (scapular region) and stored in a sterile flask free of RNase and DNase at – 20 °C. For the parasitological culture, another skin fragment as well as bone marrow and lymph node aspirates were collected and stored in sterile saline solution containing antibiotics and antifungals under refrigeration. The samples were kept at 4 °C for 24 h, then sown in biphasic culture medium containing Novy-MacNeal-Nicole medium and Schneider's insect medium supplemented with 10% fetal bovine serum (FBS), and examined weekly by optical microscopy for 1 month in search of promastigote forms of the parasite [15]. Confirmation of infection and characterization of the parasite as *L. infantum* was performed using qPCR with specific species primers [16]. After DNA extraction, the sample was amplified using the TaqMan® system on the StepOne platform™ (Applied Biosystems®, Thermo Fisher Scientific, Waltham, MA, USA). The primers LEISH-1 (5′-AACTTTTCTGGTCCTCCGGGTAG-3′) and LEISH-2 (5′-ACCCCCAGTTTCCCGCC-3′) and the probe TaqMan-MGB (FAM-5′AAAAATGGGTGCAGAAAT-3′-NFQM-3GB) were used in the qPCR, targeting a conserved region of the *L. infantum* kinetoplast DNA. The samples were amplified on the StepOne™ platform. After confirmation of infection by all proposed methodologies (DPP®, qPCR and parasitological culture), the treatment was started.

 The entire treatment was based on recommendations in the LeishVet canine leishmaniasis treatment manual [17]. Monotherapy with miltefosine was carried out according to the manufacturer’s instructions as two treatment courses with an interval of 4 months between treatments. In each treatment course, a daily dose of 2 mg/kg of the drug was administered for 28 consecutive days. In combination therapy, miltefosine was combined allopurinol that was given in two daily doses (10 mg/kg/day) during the entire treatment period, including during the 4-month interval between the two miltefosine treatments. New parasite collections were performed immediately before the start of the new course with the aim to isolate parasites in addition to those isolated prior to commencement of treatment with miltefosine (T0) and after one (T1) and two (T2) courses of miltefosine treatment. The commercial drugs milteforan™ (Virbac®), amphotericin B (generic pharmacy) and meglumine antimoniate (generic pharmacy) were used for the in vitro assays as a source of miltefosine, amphotericin B and meglumine antimoniate, respectively. The trivalent form of meglumine antimoniate (antimony potassium tartrate trihydrate; Sigma®, Sigma–Aldrich, St. Louis, MO, USA) was used in the promastigote tests against antimony. The drugs were stored as indicated on their package inserts and diluted immediately before the assays in Schneider’s culture medium until the desired concentrations were reached.

 The half maximal inhibitory concentration (IC_50_) values against T0, T1 and T2 parasites and* L. infantum* reference strain MHOM/BR/74/PP75 promastigote forms were determined using the MTT [3-(4,5-dimethylthiazol-2-yl)-2,5-diphenyltetrazolium bromide] colorimetric assay [18]. A control (no drugs added) was used for each isolate. The IC_50_ values were obtained using cell viability values for each drug. For assays against the amastigote forms of the isolates and the reference strain, the THP-1 human leukemia monocytic cell line, was used as a host. The monocytes were kept at 37 °C in a humid incubator, under an atmosphere of 5% CO_2_, in RPMI 1640 medium supplemented with 10% FBS, HEPES, and 1% antibiotic (penicillin streptomycin; Sigma®, Sigma–Aldrich). Cultures were maintained weekly until their growth reached 1 × 10^6^ cells/ml. Thereafter, THP-1 cells were seeded in 96-well plates at a density of 5 × 10^4^ cells/well in RPMI 1640 medium containing 200 nM phorbol myristate acetate (PMA). The plates were then incubated for 96 h to allow cell differentiation into adhered macrophages, with the culture medium replaced with new culture medium without PMA after 48 h. Concomitantly, the isolates and the reference strain of *L. infantum* were cultured up to 6–7 days in order to be able to inoculate cells already adhered and differentiated into macrophages. Inoculation was carried out at the ratio of 10 parasites per cell (10:1), and the wells containing the differentiated cells incubated for 4 h. The different drug concentrations were then added (in triplicate per evaluated dose) to each well and the plates were incubated for a further 48 h. After treatment, the cells were fixed with methanol and stained with DAPI to enable the intracellular amastigote count. A negative control (without treatment) was used as a 100% infection. Inhibitory activity was assessed by counting the number of intracellular amastigotes in 100 cells randomly captured from each well (×40 objective). Values were expressed as percentage of inhibition (PI) = 100 − [(*T *× 100)/*C*], where *T* represents the average number of amastigotes treated and *C* is the average number of amastigotes from the negative control [19]. The IC_50_ values were determined using PI values for each concentration of each drug. To measure the growth curve, a culture containing the isolates and the reference strain in exponential growth phase was adjusted to the concentration of 1 × 10^6^ parasites/ml and seeded in a 24-well plate (1 ml per well). The absorbance values were measured at 800 nm every 24 h for 8 days to correlate the increase in absorbance with the concentration of parasites in the culture [20]. For determination of the infectivity rates, THP-1 cells were infected with the isolates and the reference strain as previously described. After the fixing, staining and counting of 100 cells, the average number of amastigotes per cell infected with the isolates and with the reference strain were compared. The metacyclogenesis rates were determined by the negative selection methodology with peanut agglutinin (PNA) (Sigma®, Sigma–Aldrich) [21, 22]. Briefly, 6- to 7-day-old cultures of the isolates and the reference strain were collected by centrifugation (2000 *g*, 10 min) and resuspended at a concentration of 2 × 10^8^ parasites/ml in 10 ml of Schneider’s medium supplemented with 50 μg/ml PNA. The promastigotes were left at room temperature for 30 min for agglutination, following which the supernatant and the pellet were immediately collected. The pellet was resuspended once again in 10 ml Schneider’s medium supplemented with 50 μg/ml PNA, and both the pellet and supernatant were collected by centrifugation (200 *g*, 10 min). The supernatant resulting from both collections was centrifuged (2000  *g*, 10 min) to obtain the metacyclic promastigotes. The number of metacyclic promastigotes was determined by counting in a Neubauer chamber and the percentage of metacyclogenesis among the isolates was calculated by the ratio of the number of metacyclic promastigotes to the total initial promastigote population. All experiments were carried out in triplicate.

 Data normality was assessed by a Kolmogorov–Smirnov test, and the IC_50_ values was obtained with PRISM version 5 software (GraphPad Software, San Diego, CA, USA) using non-linear regression. All groups were compared using parametric one-way analysis of variance, followed by the Tukey test.

The species-specific primers used in the qPCR successfully confirmed infection by *L. infantum*, and the parasite was isolated in culture. All tests were repeated immediately before the start of a new treatment course, resulting in three different isolates: MCAN/BR/19/CG06T0 (T0), MCAN/BR/19/CG06T1 (T1) and MCAN/BR/20/CG06T2 (T2), which enabled access to the parasites at different stages throughout the dog's treatment with miltefosine plus allopurinol.

Susceptibility assays conducted with the isolates and the reference strain showed a significant increase in the IC_50_ values of the promastigote (Fig. 1a, c) (*F*_(3, 8)_ = 30.11, *P* = 0.0001) and amastigote (Fig. 1b, d) (*F*_(3, 8)_ = 27.56, *P* = 0.0001) forms, which is evidence of resistance to miltefosine and amphotericin B increasing with increasing number of treatment courses. The parasites isolated prior to treatment (T0) presented IC_50_ values against miltefosine equal to those of the control; however, these values increased after only one course of treatment (T1) (Fig. 1a, b), diverging statistically from those of the reference strain. The upward trend continued throughout the treatment, with T2 isolates presenting IC_50_ values approximately twofold higher than those of the reference strain.

**Fig. 1:**
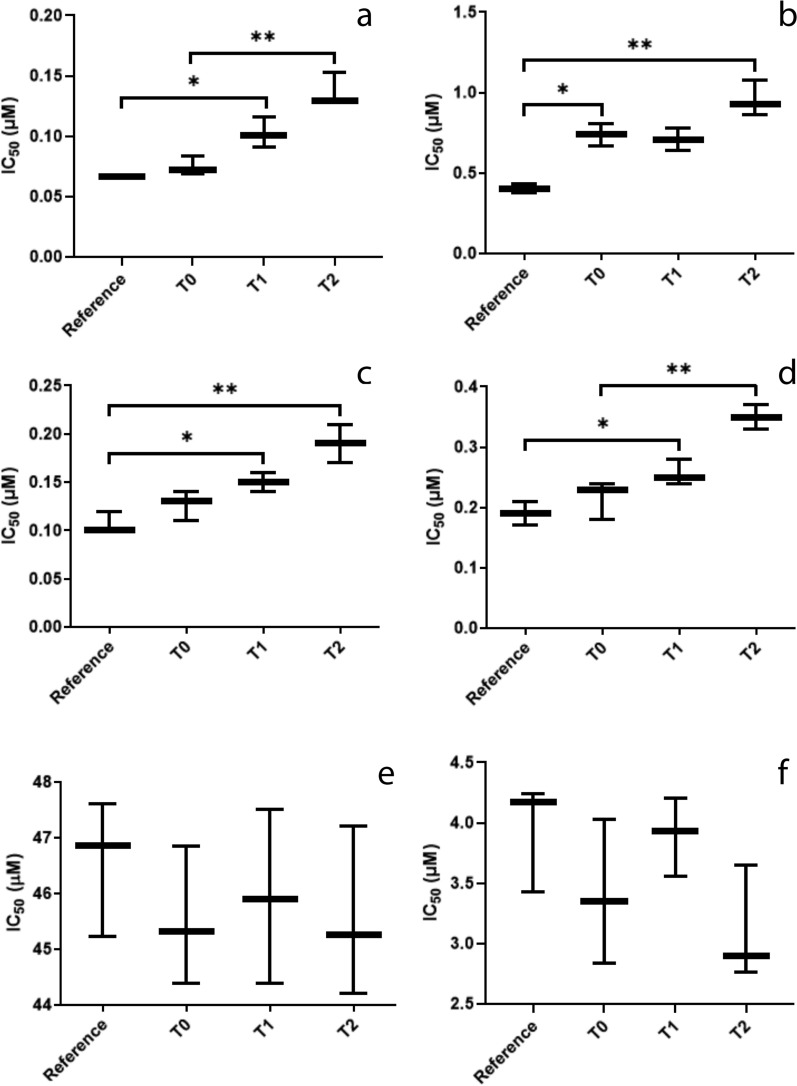
Boxplot of results of the in vitro resistance tests of *Leishmania infantum* isolates and *L. infantum* reference strain MHOM/BR/74/PP75 to the drugs tested. **a**, **b** IC_50_ values of the promastigote (**a**) and amastigote (**b**) forms of the isolates and reference strain against miltefosine. **c**, **d** IC_50_ values of promastigote (**c**) and amastigote (**d**) forms of the isolates and reference strain against amphotericin B. **e**, **f** IC_50_ values of promastigote (**e**) and amastigote (**f**) forms of the isolates and reference strain against meglumine antimoniate. Asterisks indicate statistical significance at *P < 0.05 and **P < 0.01. Abbreviations: IC_50_, Half maximal inhibitory concentration; T0, prior to commencement of treatment with miltefosine; T1, T2, after 1 and 2 courses of miltefosine treatment, respectively

The same pattern was observed in the parasites treated with amphotericin B, where an increase in resistance to the drug was verified throughout the treatment courses (Fig. 1c, d). In the promastigote forms of the parasites (Fig. 1c), the IC_50_ values of the isolates before the dog’s treatment with miltefosine plus allopurinol (T0) was already higher than that of the reference strain (*F*_(3, 8)_ = 17.74, *P* = 0.0007); this was also observed at T1 in the assays with amastigote forms (Fig. 1d) (*F*_(3, 8)_ = 25.96, *P* = 0.0002). No statistical difference was found between the IC_50_ values of the T0, T1, and T2 isolates against meglumine antimoniate.

Results of the growth curve of the isolates in culture medium showed no difference between the number of parasites or cell proliferation rate. The infectivity rate was higher in the reference strain (Fig. 2). The cells infected with the reference strain had an average of six amastigotes per cell, which was more than the number in T0 (< 4 amastigotes per cell) and T1 and T2 (both with < 2 amastigotes per cell) isolates (*F*_(3, 8)_ = 54.76, *P* < 0.0001).

**Fig. 2:**
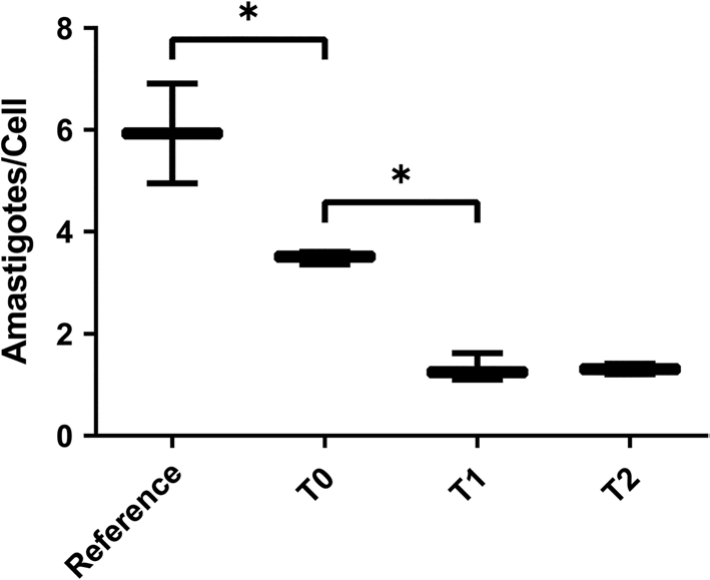
Boxplot of average number of amastigotes per THP-1 cell infected with the *L. infantum* reference strain (MHOM/BR/74/PP75), parasites isolated from the dog before treatment (T0), after 1 course of treatment (T1) and after 2 courses of treatment (T2). Asterisk indicates statistical significance at *P < 0.05

The metacyclogenesis rates showed a clear upward trend with increasing treatment, although the differences were not statistically significant. Parasite samples isolated from the dog before treatment with miltefosine plus allopurinol showed an average of 2 × 10^4^ parasites/ml. After one course of treatment, this number increased to about 3 × 10^4^ parasites/ml, ultimately reaching approximately 5 × 10^4^ parasites/ml after two treatment courses.

A strong linear correlation (*R*^2^ = 0.87) was observed between the number of treatment courses with miltefosine plus allopurinol to which the dog was subjected and the increase in the IC_50_ values of *L. infantum* isolates against miltefosine. One explanation of these results is that the treatment of dogs with miltefosine is not effective in achieving a complete elimination of the parasites [5–7]. Those parasites that remain in the dog are exposed to subtherapeutic doses, resulting from the long half-life of this drug [23], which drive a selection of resistant parasites.

There is general consensus that most dogs treated with miltefosine respond well to treatment, with the parasite load greatly reduced for periods longer than 4 months [5]. However, in a minority of cases, the treatment is not successful, and the dog becomes a favorable environment for the emergence of resistant parasites, as observed in the present study. It is well established that the therapeutic success of the treatment and the clinical course of the disease are closely linked to the profile of the dog's immune response [25]. Dogs that have a Th1-polarized immune response have a better prognosis than those that have a Th2 immune response. A humoral response profile (Th2) is not efficacious in combating the intracellular parasite, which leads to the need for successive therapeutic rounds with the drug, thereby facilitating the emergence of resistance.

Studies have also shown that resistance to miltefosine remains constant even after passage through sand flies [26] and successive in vitro passages [24].This finding, combined with the use of miltefosine therapy to treat CanL in endemic areas and intense zoonotic transmission, and coupled with the knowledge that the dog can be a source of vector infection for *L. infantum* even weeks after the end of treatment, despite being clinically cured [8], may aggravate the problem involving the emergence of resistant parasites, since the dog can become infected with parasites that have already come into contact with the drug and, consequently, already present high resistance to it. As such, it is fundamental that not only uninfected dogs but also infected dogs under treatment are protected through the use of insecticides and repellents [17].

The isolates analyzed showed the acquisition of resistance not only to one of the drugs they had contact with during the treatment of the dog (miltefosine), but also to amphotericin B. This phenomenon, called cross-resistance, is well established in species of the genus *Leishmania* and involves several drugs [27–29]. Mondelaers et al. [27] reported clear cross-resistance between miltefosine and amphotericin B, corroborating the findings of the present study. In our analyses, a strong linear correlation (*R*^2^ = 0.83) was observed between the number of treatment courses with miltefosine plus allopurinol and the increase in the IC_50_ values of *L. infantum* isolates against amphotericin B. Acquisition of resistance to amphotericin B was similar to that to miltefosine, with IC_50_ values already higher at T1 than at T0, reaching values about of 1.8-fold higher than those at T0 after two courses of treatment with miltefosine (T2).

The possible cross-resistance between miltefosine and amphotericin B is a concern, since amphotericin B is one of the most commonly used drugs to treat VL in humans. Thus, parasites resistant to both drugs could be transmitted to other dogs and, eventually, to humans, also considering that resistance is maintained even after passage through sand flies [26]. Amphotericin B, in its liposomal formulation, is used to treat VL in pregnant women, children aged < 1 year, individuals aged > 50 years, those with comorbidities and those who are HIV positive [30]. All of of these groups are considered at risk for the disease and require less toxic and a more effective treatment. The effectiveness of amphotericin B can be reduced by the emergence of parasites resistant to the drug.

Additionally, allopurinol (used in combination with miltefosine in the dog treated herein) is a leishmaniostatic drug [31, 32]. There is a previous report of cross-resistance between meglumine antimoniate and allopurinol, but not between these drugs and miltefosine or amphotericin B [28]. These data suggest that the increased resistance of the parasites to amphotericin B and miltefosine observed in our study is due to the contact of the parasites with miltefosine—and not allopurinol; however, further research is required.

There did not appear to be any significant changes in the parasite fitness parameters associated with the acquisition of resistance. While some studies demonstrate that the acquisition of resistance is followed by increased rates of infectivity, proliferation and metacyclogenesis [12], other studies point out that the parasite shows a decrease in some of these parameters in exchange for resistance, in a type of metabolic exchange currency [13, 26]. In our study, the metacyclogenesis rates showed an upward trend that corresponded with an increasing number of treatment courses with miltefosine plus allopurinol; however, the trend was not statistically significant. Some authors consider the metacyclogenesis rate to be one of the most important parameters that determine the virulence of a strain [14], since a larger number of parasite infective forms in the vectors may favor the establishment of the infection in the vertebrate host [33].

In conclusion, the data obtained in this study indicate increased *L. infantum* resistance to miltefosine and amphotericin B after the treatment of a dog with miltefosine plus allopurinol. Considering that we used strains isolated from a single dog, further studies are needed to better understand the impact of treating dogs with miltefosine (alone or in combination with allopurinol) on the emergence of resistant strains. Ideally, these studies should include a larger number of *L. infantum* strains isolated from dogs, with varied immune response profiles and submitted to different treatment regimes.

## Data Availability

Not applicable.

## Abbreviations

CanL: Canine leishmaniasis
VL: Visceral leishmaniasis
